# Falls Among Older Adults in Türkiye: A Nationwide Analysis of Emergency Medical Service Data

**DOI:** 10.3390/jcm15187239

**Published:** 2026-09-17

**Authors:** Mehmet Alptekin Acar, Muhammed Saltuk Deniz, Büşra Can, Ayşenur Yetik Aras, Fatma Yorulmaz, Aslı Tufan

**Affiliations:** 1 Department of Internal Medicine, Marmara University Pendik Training and Research Hospital, Istanbul 34899, Türkiye; 2 General Directorate of Emergency Health Services, Republic of Türkiye Ministry of Health, Ankara 06800, Türkiye; m.deniz@saglik.gov.tr (M.S.D.); aysenur.yetik@saglik.gov.tr (A.Y.A.); 3 Division of Geriatrics, Department of Internal Medicine, Faculty of Medicine, Marmara University, Istanbul 34854, Türkiye; alpabusra@hotmail.com (B.C.); aslitufan@yahoo.com (A.T.); 4 Department of Emergency Medicine, Ministry of Health Ankara Etlik City Hospital, Ankara 06170, Türkiye; drfatmakose@hotmail.com

**Keywords:** accidental falls, aged, emergency medical services, patient transfer, prehospital care

## Abstract

**Background/Objectives:** Many ambulance-attended falls among older adults are not represented in hospital-based datasets. We used emergency medical service (EMS) records to describe residential and non-residential falls and identify factors associated with non-residential fall location and hospital transfer. **Methods:** We retrospectively analyzed records from Türkiye’s 112 EMS system from January 2023 through October 2025. The analytic sample comprised 685,666 records; no age-eligible records were excluded for missing study variables. Fall-location associations were examined using chi-square tests, Cramér’s V, and multivariable logistic regression; a multivariable model examined hospital transfer. **Results:** Residential falls accounted for 406,183 records (59.2%), increasing from 49.6% at ages 65–74 to 61.9% at 75–84 and 71.1% at ≥85 years, and occurring more often among women than men (65.4% vs. 50.8%). Adjusted odds ratios (ORs) for non-residential location were higher for daytime occurrence (08:00–16:59: OR = 2.871) and male sex (OR = 1.761) and lower with advancing age (≥85 years: OR = 0.424). Hospital transfer occurred in 527,881 records (77.0%). Adjusted odds were higher for yellow-coded (OR = 11.063) and red-coded (OR = 8.053) cases, lower for non-residential location (OR = 0.268) and age ≥ 85 years (OR = 0.739), and higher outside Istanbul (OR = 1.999). **Conclusions:** This is the first nationwide prehospital analysis of falls among older adults in Türkiye. Residential falls predominated, while fall location and hospital transfer showed demographic, temporal, triage, and geographic associations. These findings may inform research on fall prevention, prehospital assessment, and referral pathways.

## 1. Introduction

Falls are among the most frequent and consequential adverse health events affecting older adults [1,2]. Approximately one in three community-dwelling adults aged ≥65 years falls at least once each year, and both incidence and severity rise with advancing age [1,2,3]. A fall in later life is often a sentinel event marking the onset of functional decline, and recurrent falls—including those that produce no visible injury—are associated with increased morbidity and mortality [4]. Falls and fall-related injuries increase the risk of subsequent nursing home admission [5], and fear of falling further restricts mobility and social participation [6]. Fall-related hospital admissions, emergency department visits, rehabilitation, and long-term care account for a substantial share of health care expenditure in aging populations [5,7].

Emergency medical services (EMS) occupy a distinctive vantage point in the care of older adults who fall, because ambulance crews are frequently the first clinicians to assess the patient [8,9,10]. A considerable proportion of EMS-attended falls are managed on scene and never reach the hospital, so hospital-based registries systematically underestimate the magnitude of the problem [9,10]. Prehospital records therefore capture a segment of the fall burden that remains invisible to hospital-centered surveillance.

Two features of the prehospital encounter are clinically informative yet rarely examined at the population scale. The first is where the fall occurred. Indoor falls tend to cluster among the oldest old and among individuals with greater functional limitation, whereas outdoor falls more often involve relatively active older adults exposed to environmental hazards such as uneven surfaces or traffic [11,12]. The second is the decision to transfer the patient to the hospital, which is made under time pressure and with limited clinical information [8,10]. Transporting a frail older adult may expose the patient to prolonged waiting and disruption of care continuity, whereas not transporting a patient who needs hospital-level care risks missed diagnoses and delayed treatment. Previous studies suggest that transport disposition is associated with clinical severity, age, sex, fall location, field triage urgency, and geographic or service context [9,10].

Characterizing these encounters at a national scale may have several practical implications. Patterns in fall location across demographic groups may help identify settings and populations in which preventive strategies warrant greater attention. Similarly, examining factors associated with hospital transfer may inform future evaluation of prehospital assessment practices, geriatric fall training, and referral criteria for older adults who may not require emergency department care. Describing the temporal and geographic distribution of EMS-attended falls may also support service planning and the development of appropriate follow-up pathways. Although these applications require prospective evaluation, nationwide EMS data provide an empirical basis for informing such clinical and organizational strategies.

Türkiye’s 112 Emergency Medical Services system is a centralized, government-administered ambulance dispatch network covering all 81 provinces, and it records every ambulance-attended event regardless of eventual transport [13]. We used these national records from January 2023 through October 2025 to describe the distribution of residential and non-residential falls among adults aged ≥65 years; assess their bivariate associations with patient, temporal, geographic, and triage characteristics; and identify multivariable factors associated with non-residential fall location and hospital transfer.

## 2. Materials and Methods

### 2.1. Study Design and Setting

We conducted a retrospective, observational, national case-level analysis of fall records among adults aged ≥65 years collected through Türkiye’s 112 EMS system from January 2023 through October 2025. Records for 2023 and 2024 cover the full calendar year. Records for 2025 cover January to October only, because data for November and December 2025 had not yet been consolidated in the national system at the time of extraction. The study period therefore comprises 34 consecutive months. Year of incident was not entered into any inferential analysis, and the incomplete final year affects only the descriptive distribution of records by year (Table 1). The system operates under the Republic of Türkiye Ministry of Health and serves urban and rural populations across all 81 provinces through regionally distributed ambulance stations [13]. Each response generates a standardized electronic case record containing demographic, clinical, temporal, and geographic fields. Because every ambulance-attended event is captured regardless of whether the patient is transported, the system provides a view of falls that could not be obtained from hospital-based registries alone. The manuscript was prepared with reference to the Strengthening the Reporting of Observational Studies in Epidemiology (STROBE) reporting recommendations [14] (File S1).

As of 2025, Türkiye had a total population of 86,092,168, including 9,583,059 adults aged ≥65 years (11.1%) [15,16]. Within the population aged ≥65 years, 55.3% were women and 44.7% were men; 62.9% were aged 65–74 years, 29.3% were aged 75–84 years, and 7.8% were aged ≥85 years [15]. These figures provide demographic context for the national population served by the 112 EMS system and for the EMS-attended fall records analyzed in the present study.

The unit of analysis was the individual EMS case record, not the individual patient. Recurrent presentations by the same person were not linked, and each record was treated as an independent case; no patient-level deduplication was performed. Counts throughout this report therefore refer to case records rather than to unique individuals.

### 2.2. Data Source

All data were obtained from the Emergency Health Automation System (Acil Sağlık Otomasyon Sistemi) maintained by the General Directorate of Emergency Health Services of the Republic of Türkiye Ministry of Health. Records are generated at the point of care by ambulance crews during on-scene assessment and, where applicable, during transport. Each record contains structured fields for demographics (age, sex), incident characteristics (date, year, time of day, fall location, settlement type, city), clinical assessment (consciousness status, triage category), and case disposition (transfer status). No supplementary sources—such as hospital admission records, outpatient follow-up data, or mortality registries—were linked to the EMS records.

### 2.3. Study Population

**Figure 1:**
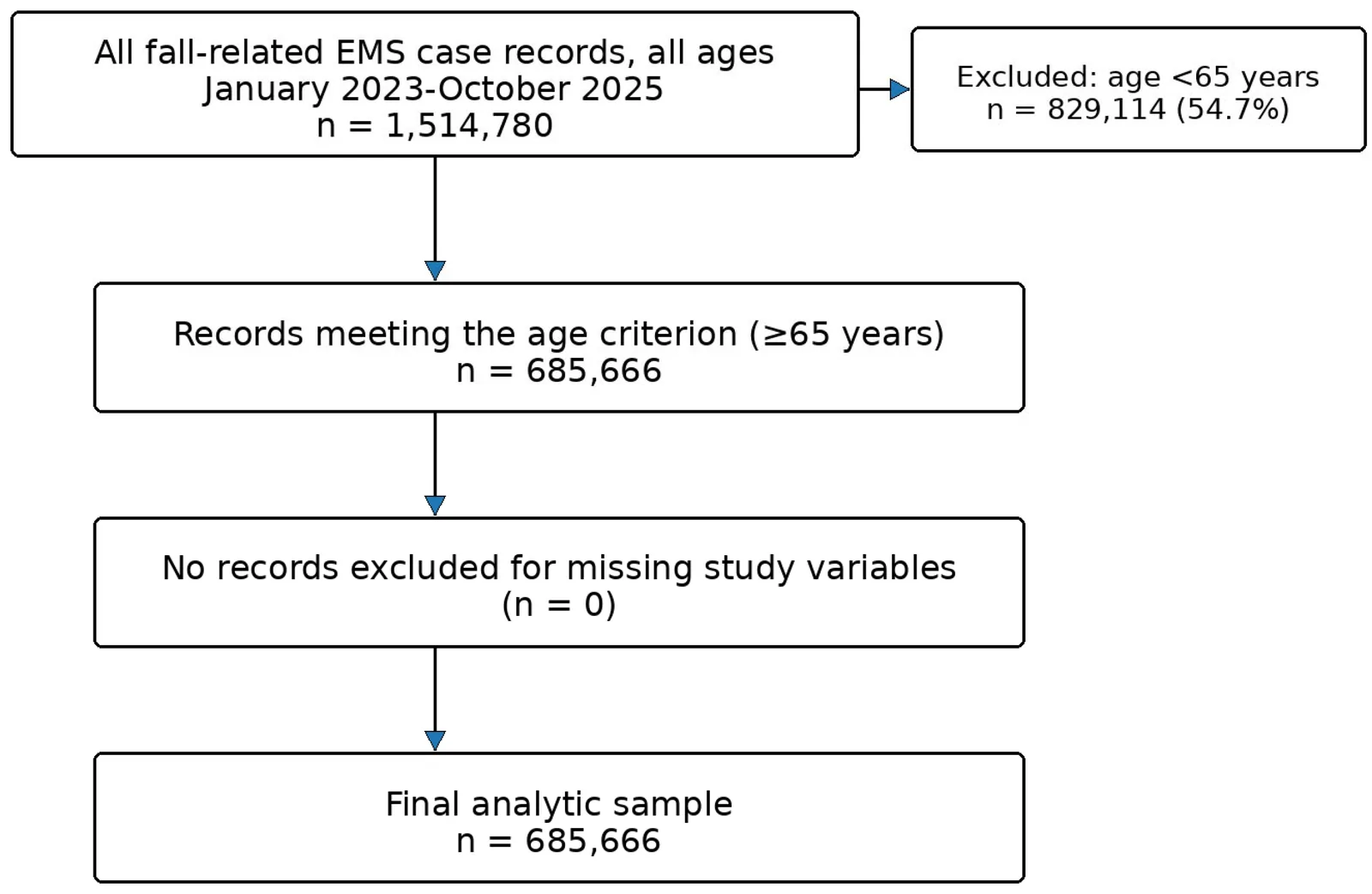
Flow of emergency medical services fall case records through study inclusion. EMS, emergency medical services. Records involving individuals aged <65 years were excluded on the basis of the prespecified age criterion; no records meeting the age criterion were excluded because of missing study variables.

During January 2023 through October 2025, the database contained 1,514,780 EMS case records coded as falls across all ages. The study population was restricted to records involving adults aged ≥65 years. Accordingly, 829,114 records involving individuals aged <65 years were excluded, leaving 685,666 records that met the age criterion. No records meeting the age criterion were excluded because of missing values in the study variables (age, sex, fall location, triage category, transfer status, consciousness status, settlement type, or city), and no imputation was required. No further exclusions were applied on the basis of clinical severity, disposition, or geographic location. The final analytic sample therefore comprised 685,666 EMS case records. The selection process is summarized in Figure 1.

### 2.4. Variables and Definitions

Fall location was the primary grouping variable and was derived from the location field recorded by ambulance crews. For analysis, the operational EMS location codes were grouped into residential and non-residential categories. These categories represent administrative/operational groupings within the EMS dataset and should not be interpreted as a direct indoor–outdoor classification. The residential category comprises falls occurring at home, in a nursing home, hotel, prison, or dormitory. The non-residential category comprises falls at health care facilities, on the street, in a vehicle, on a highway, in field or terrain settings, at a workplace, and in other non-residential locations. Several residential categories are institutional, and several non-residential categories are indoor. Records coded as health care facility include both falls occurring within a health care facility and records in which the individual had already reached a facility before EMS contact. Accordingly, for this category, the recorded location may represent the point of EMS contact rather than the actual site of the fall. These records therefore cannot be interpreted uniformly as falls occurring within health care facilities.

Time of incident was categorized as 00:00–07:59, 08:00–16:59, or 17:00–23:59, broadly distinguishing nocturnal and early-morning hours, standard daytime hours, and the evening period. Age was recorded as a continuous variable and grouped into 65–74, 75–84, and ≥85 years. These categories were selected to align with the age strata used in Turkish national population statistics [15], thereby facilitating comparison with the reference population. The first two categories comprise equal 10-year intervals, while the ≥85-year category was retained as an open-ended group consistent with the national reporting framework. Sex was recorded as female or male. Year of incident was recorded as 2023, 2024, or 2025. Consciousness status was recorded by the ambulance crew in the standardized 112 EMS case record as a categorical field rather than as a Glasgow Coma Scale (GCS) score or another formal neurological scale. For the present analysis, the recorded field was dichotomized as normal (alert and oriented) versus abnormal (any documented alteration in consciousness). Because the dataset did not contain a standardized numerical consciousness score, this variable was treated as an operational EMS assessment rather than a validated measure of neurological status. City was coded as Istanbul versus all other provinces. Istanbul was analyzed separately because it represents a marked demographic outlier within Türkiye: in 2025, Istanbul had 15,754,053 residents, accounting for 18.3% of the national population, and a population density of 2943 persons/km^2^ compared with 112 persons/km^2^ nationally [16]. The next most densely populated province had 633 persons/km^2^, further illustrating the exceptional population concentration in Istanbul [16]. This dichotomization was intended to distinguish this highly concentrated metropolitan setting from the remainder of the country. Nevertheless, the non-Istanbul category combines 80 provinces into a single comparator and therefore cannot capture province-level demographic or health-system variation; accordingly, this variable should be interpreted as a broad geographic contrast rather than as a measure of specific regional health-system differences. Settlement type was classified as urban or rural according to the administrative designation recorded in the system. Triage category was recorded as green, yellow, or red according to the operational triage coding used by the 112 EMS system; these codes reflect the field priority assignment made by the crew at the scene and are not formal injury severity scores or standardized clinical acuity scales. Case outcome was the disposition recorded at the conclusion of the response, retained as documented in the system: hospital transfer, inter-hospital transfer, transfer refusal, transfer to home, transfer for medical examination, on-scene intervention, death on scene, transfer to morgue, and other.

### 2.5. Outcome Definition

Hospital transfer was defined as a binary operational outcome based on the disposition field recorded in the EMS dataset. Case records coded as “hospital transfer” constituted the outcome group, whereas all other recorded disposition codes—including inter-hospital transfer, transfer refusal, transfer to home, transfer for medical examination, on-scene intervention, death-related outcomes, and other dispositions—constituted the reference group. This dichotomization was intended to distinguish records specifically coded as hospital transfer from all other documented EMS dispositions and does not imply that the heterogeneous categories within the reference group are clinically or operationally equivalent.

Inter-hospital transfer was retained as a distinct disposition code and was not combined with hospital transfer in the primary model. Because inter-hospital transfers represented a substantial proportion of records and a distinct disposition context, a sensitivity analysis was performed in which all records coded as inter-hospital transfer were excluded. After exclusion of 83,074 records, the hospital-transfer model was refitted in 602,592 records using the same covariates and reference categories as in the primary analysis.

### 2.6. Statistical Analysis

Descriptive statistics are reported as frequencies and percentages, overall and separately for residential and non-residential falls. Bivariate associations between fall location and each categorical variable—time of incident, age group, sex, city, triage category, settlement type, and consciousness status—were assessed with the Pearson chi-square test. Because the large sample size could render small differences statistically significant, Cramér’s V was calculated for each bivariate comparison to quantify association strength. Effect size was interpreted using Cohen-derived thresholds adjusted for contingency-table degrees of freedom: for df = 1, V values of 0.10, 0.30, and 0.50 indicate small, medium, and large associations, respectively; for df = 2, the corresponding thresholds are 0.07, 0.21, and 0.35 [17]. Statistical significance and effect magnitude were interpreted separately.

To examine factors independently associated with fall location, a binary logistic regression model was fitted with non-residential location as the outcome and residential location as the reference category. Independent variables were time of incident, triage category, age group, sex, city, and settlement type. Reference categories were 00:00–07:59, green triage code, 65–74 years, female sex, Istanbul, and urban settlement, respectively. Results are reported as odds ratios (ORs) with 95% confidence intervals (CIs) and *p*-values; an OR greater than 1 indicates higher odds of a non-residential location relative to the reference category.

We constructed a binary logistic regression model for hospital transfer (hospital transfer versus all other dispositions). Independent variables were time of incident, fall location, triage category, age group, sex, city, and settlement type. Consciousness status was retained for descriptive and bivariate analyses but was not included in either multivariable model. The available variable was a binary operational EMS field that grouped any documented alteration in consciousness into a single abnormal category and did not quantify the degree of neurological impairment. We therefore chose not to use this coarse operational field as a clinical severity covariate in the adjusted models. This was an analytic choice rather than a technical constraint and should not be interpreted as indicating that consciousness status is clinically unimportant. Results are reported as odds ratios (ORs) with 95% CIs and *p*-values; an OR greater than 1 indicates higher odds of hospital transfer relative to the reference category. Because hospital transfer was a common outcome in this population, odds ratios are reported as measures of association and are not interpreted as risk ratios or as relative changes in absolute probability.

Both multivariable models included all 685,666 case records; no observations were dropped from either analysis. Given the sample size (n = 685,666), even small differences in proportions were expected to reach conventional statistical significance. Interpretation therefore emphasizes the direction and magnitude of effects rather than *p*-values alone. Very small *p*-values are reported as *p* < 0.001, and the significance threshold was *p* < 0.05. All associations are reported as statistical associations and should not be interpreted as causal. Analyses were performed with IBM SPSS Statistics version 26 (IBM Corp., Armonk, NY, USA). A sensitivity analysis excluding all records coded as inter-hospital transfer was performed to assess whether inclusion of this distinct disposition within the reference group materially affected the estimates from the primary hospital-transfer model. The model was refitted using the same covariates and reference categories as in the primary analysis.

### 2.7. Ethical Considerations

Ethical approval was granted by the Non-Interventional Clinical Research Ethics Committee of Ankara Yıldırım Beyazıt University Yenimahalle Training and Research Hospital (decision date: 30 April 2026; registration number: E-2026-23). The study was reviewed under the title “Geriatrik Hastalarda Düşme Vakalarının Ambulans Müdahaleleri ile Analizi” and was approved unanimously. The requirement for informed consent was waived because of the retrospective design and the use of anonymized, routinely collected EMS data. Patient identifiers were not included in the analytic dataset, and no attempt was made to re-identify individual patients.

## 3. Results

Among 685,666 fall case records, 406,183 (59.2%) occurred in residential and 279,483 (40.8%) in non-residential settings (Table 1). Women accounted for 394,395 case records (57.5%), and 157,243 (22.9%) involved adults aged ≥85 years. Most falls occurred during daytime hours (403,925; 58.9%), and most were assigned a yellow triage code (450,064; 65.6%). Consciousness was recorded as normal in 662,387 case records (96.6%).

Hospital transfer was the most common disposition overall (527,881; 77.0%) and was more frequent after residential than non-residential falls (93.3% vs. 53.3%); inter-hospital transfer and transfer to home were concentrated among non-residential falls (29.1% and 10.9%, respectively) (Table 2). Within the residential category, 389,764 falls (96.0%) occurred at home and 12,445 (3.1%) in nursing homes; within the non-residential category, records coded as health care facility (124,521; 44.6%) and street (114,456; 41.0%) predominated (Table 3).

Percentages are column percentages within each fall-location category. Disposition categories are reported as documented in the EMS dataset. In the primary hospital-transfer regression model, records coded as “Transfer—hospital” constituted the outcome group, and all remaining disposition categories constituted the reference group. Records coded as inter-hospital transfer were excluded in the sensitivity analysis.

Fall location differed statistically across every characteristic examined (*p* < 0.001 for all), but effect sizes varied substantially (Table 4). The largest associations were observed for age group (V = 0.171), sex (V = 0.147), and time of incident (V = 0.142), each corresponding to a small association under the df-adjusted thresholds. The proportion of residential falls increased from 49.6% among adults aged 65–74 years to 61.9% among those aged 75–84 years and 71.1% among those aged ≥85 years; it was also higher among women than men (65.4% vs. 50.8%) and highest during 00:00–07:59 (77.6%). In contrast, associations with settlement type (V = 0.033), triage category (V = 0.031), city (V = 0.023), and consciousness status (V = 0.010) were below the corresponding threshold for a small effect despite statistical significance.

In the multivariable fall-location model, the largest adjusted associations were observed for time of incident, age, and sex (Table 5). Compared with 00:00–07:59, odds of a non-residential location were higher during 08:00–16:59 (OR, 2.871; 95% CI, 2.820–2.923) and 17:00–23:59 (OR, 2.390; 95% CI, 2.345–2.437). Male sex was associated with higher odds of a non-residential location than female sex (OR, 1.761; 95% CI, 1.743–1.778). In contrast, odds were lower with advancing age (75–84 years: OR, 0.624; 95% CI, 0.617–0.631; ≥85 years: OR, 0.424; 95% CI, 0.418–0.430). Smaller adjusted associations were observed for non-Istanbul location (OR, 1.140; 95% CI, 1.124–1.157), rural settlement (OR, 0.793; 95% CI, 0.784–0.803), yellow triage (OR, 0.897; 95% CI, 0.886–0.907), and red triage (OR, 0.939; 95% CI, 0.921–0.957); *p* < 0.001 for all.

In the multivariable hospital-transfer model, triage category and fall location showed the largest associations with hospital transfer (Table 6). Relative to green-coded cases, odds of transfer were higher for yellow-coded (OR, 11.063; 95% CI, 10.858–11.271) and red-coded cases (OR, 8.053; 95% CI, 7.780–8.337; *p* < 0.001 for both). Non-residential falls had lower odds of transfer than residential falls (OR, 0.268; 95% CI, 0.264–0.273; *p* < 0.001). Odds of transfer decreased with age (75–84 years: OR, 0.863; 95% CI, 0.846–0.880; ≥85 years: OR, 0.739; 95% CI, 0.722–0.756; *p* < 0.001 for both). Cases outside Istanbul had higher adjusted odds of hospital transfer than cases in Istanbul (OR, 1.999; 95% CI, 1.953–2.045; *p* < 0.001). Smaller associations were observed for rural settlement (OR, 1.165; 95% CI, 1.140–1.191), male sex (OR, 1.158; 95% CI, 1.138–1.179), and the 17:00–23:59 period (OR, 1.124; 95% CI, 1.088–1.161), whereas the 08:00–16:59 period was associated with marginally lower odds than 00:00–07:59 (OR, 0.959; 95% CI, 0.931–0.988; *p* = 0.006).

In the sensitivity analysis excluding 83,074 records coded as inter-hospital transfer, 602,592 records remained for analysis. The direction of the principal associations was consistent with the primary hospital-transfer model, although the magnitudes of the estimates changed. Non-residential location remained associated with lower odds of hospital transfer (OR, 0.181; 95% CI, 0.178–0.185). Yellow-coded cases (OR, 9.681; 95% CI, 9.498–9.868) and red-coded cases (OR, 7.285; 95% CI, 7.033–7.546) remained associated with higher odds of hospital transfer than green-coded cases, with the estimate for yellow remaining higher than that for red. The principal associations therefore remained directionally consistent after exclusion of inter-hospital transfers.

## 4. Discussion

In this nationwide analysis of 685,666 EMS-attended fall case records among adults aged ≥65 years, three findings stand out. First, falls in older adults attended by ambulance occurred predominantly in residential settings, and the adjusted fall-location model showed the largest associations with time of incident, age, and sex. Second, hospital transfer followed just over three-quarters of encounters and was associated most strongly with the field triage category assigned at the scene and with the location of the fall, while the odds of transfer fell as age increased. Third, the disposition decision varied by geography, with cases outside Istanbul showing higher adjusted odds of hospital transfer than cases in Istanbul. Because these are EMS-attended events recorded at the point of care, they include the substantial group of falls that are resolved on scene and never appear in hospital-based data.

The majority of falls identified in this study occurred within residential settings. Prior studies examining indoor falls have reported associations with poorer functional status and frailty-related characteristics among older adults [11,12]. However, our residential/non-residential classification is not equivalent to an indoor/outdoor distinction, and frailty, functional status, mobility, and homebound status were not available in the present EMS dataset. These factors should therefore be regarded only as possible contextual explanations and cannot be evaluated as mechanisms underlying the residential fall pattern observed in this study. Emerging digital fall-detection technologies, including motion- and sensor-based systems, are being investigated for use in aged-care settings [18]. Such tools may support earlier detection and response to fall incidents, although further clinical validation is needed.

In alignment with previous literature [19], women accounted for a greater proportion of fall-related EMS records than men in the present study. Previous studies have discussed biological and functional differences, including differences in muscular strength and physical performance, as possible contributors to sex-related variation in fall patterns [19]. However, these characteristics were not measured in the present dataset, and the mechanisms underlying the observed sex difference therefore cannot be determined from our data.

Non-residential falls accounted for a greater proportion of records during daytime and evening hours than during late-night and early-morning hours, and their proportion decreased with advancing age. Non-residential falls also constituted a greater proportion of records among men than among women. Although our residential/non-residential classification is not directly equivalent to an indoor/outdoor distinction, these demographic patterns are broadly consistent with previous studies reporting that outdoor falls occur relatively more often among men and among adults at the lower end of the older age range [11]. Previous studies have also described outdoor fallers as relatively more physically active and healthier than indoor fallers [11]; however, physical activity, functional capacity, frailty, and comorbidity were not available in the present dataset, and these characteristics therefore cannot be inferred from the present EMS data.

The multivariable analysis reinforced the principal fall-location patterns observed in the bivariate analyses. After adjustment, time of incident, age, and sex showed the largest associations with non-residential location: odds were higher during daytime and evening hours and among men, and progressively lower across older age groups. City, settlement type, and triage category showed smaller adjusted associations. These findings should be interpreted within the operational residential/non-residential classification used in the EMS dataset, which is not directly equivalent to an indoor/outdoor distinction (Table 5).

A study conducted in İzmir, Türkiye, analyzing 112 emergency calls, indicated that ambulance interventions for geriatric patients were found to be more frequent in urban areas, peaking between 08:00 and 17:00, which aligns with our data [20]. This pattern likely stems from the high incidence of falls (40%) occurring during ambulation [21].

For context, prior research has indicated that approximately 80% of fall-related emergency calls result in hospital transport [9]. Hospital transport in the cited literature represents a broader concept than the administrative hospital-transfer category used as the outcome in the present study. While the majority of these patients require clinical evaluation and treatment, a subset is not transported due to the absence of injury, the presence of only minor trauma, or the patient’s refusal of further medical intervention [9].

Decision-making for hospital transport is notably more complex in geriatric fall cases than in clear-cut emergencies such as cardiac arrest or major trauma [22]. Consequently, ambulance personnel and prehospital providers may require additional clinical support in this domain. One such initiative, the Support and Assessment for Fall Emergency Referrals (SAFER), provides decision-making tools that guide staff in identifying suitable patients for fall service referrals versus hospital conveyance by using vital signs with environmental risk assessments [23].

The principal hospital-transfer associations remained directionally consistent after exclusion of inter-hospital transfers, although their magnitudes changed, supporting the robustness of the directional findings to exclusion of this distinct disposition category. In the present study, “hospital transfer” refers specifically to the administrative EMS disposition category defined in the Methods and should not be interpreted as synonymous with the broader concept of hospital conveyance.

The adjusted odds of hospital transfer were higher for both yellow- and red-coded cases than for green-coded cases, and the field triage category was strongly associated with hospital transfer. This association should not be interpreted as evidence that the assigned triage category directly determined the transport disposition. The odds ratio for yellow-coded cases was higher than that for red-coded cases; however, the present dataset does not allow the clinical or operational reasons for this pattern to be determined. Accordingly, these findings should be interpreted as associations between the field priority codes recorded at the scene and the documented EMS disposition rather than as a direct ordinal relationship between triage severity and hospital transfer (Table 6).

Cases outside Istanbul had higher adjusted odds of hospital transfer than cases in Istanbul. However, because the non-Istanbul category combines 80 provinces into a single comparator, this finding should be interpreted only as a broad geographic association. Unmeasured regional characteristics may contribute to the observed association, but these mechanisms could not be evaluated in the present dataset (Table 6).

One of the more counterintuitive findings was that the adjusted odds of transfer were progressively lower with advancing age. A similar inverse age-related association has been reported among older adults with major trauma, in whom increasing age was associated with a reduced likelihood of ambulance transport to a trauma center [24]. The underlying factors cannot be determined from our data and may involve unmeasured clinical or patient-level factors, including patient or caregiver preferences, differences in clinical presentation, injury characteristics, or other characteristics not captured in the EMS dataset. Further research incorporating more detailed clinical information is needed to clarify this association (Table 6).

Men had modestly higher adjusted odds of hospital transfer than women (OR, 1.158; 95% CI, 1.138–1.179; Table 6). This direction runs counter to several earlier reports, and the discrepancy is worth stating plainly rather than smoothing over. Cox et al. found that women made up 60% of ambulance-attended fallers and were more often conveyed [25], and other work has described women as more susceptible to severe fall-related injury, a pattern usually attributed to the higher prevalence of osteoporosis and the consequent risk of fracture [21]. Two considerations bear on the difference. First, the quantities are not equivalent: those figures describe the crude composition of the transported population, whereas our estimate is the adjusted odds of transfer for an individual encounter, conditional on triage category, fall location, age, and geography. Women accounted for 57.5% of the case records in our sample, so the absolute number of women with records coded as hospital transfer was larger even though the adjusted odds of the hospital-transfer outcome were slightly higher among men; a crude comparison in our data would therefore not necessarily reproduce the direction reported here. Second, men more often fell in non-residential settings, and male sex has been associated with fall-related head injuries [19]; residual differences in injury pattern may therefore persist after adjustment for fall location. Our data cannot distinguish between these explanations, since neither injury type nor bone health was recorded, and because no sex-by-location interaction or location-stratified model was fitted, the association should not be read separately for residential and non-residential falls (Table 6).

A retrospective analysis of geriatric fall patients in Australia (2010–2017) reported that 79% of emergency medical service encounters resulted in hospital conveyance [25]. Although numerically close to the 77.0% of records coded as hospital transfer in our study, these proportions should not be considered directly equivalent because they reflect different operational definitions and health-system contexts. In that study, the predictors of transport included female sex, comorbidity, and fulfillment of major trauma triage criteria. As noted above, the opposite direction for sex in our model may reflect the distinction between crude and adjusted comparisons; differences in prehospital triage protocols and in the composition of attended cases may also contribute to the variation observed across settings.

Current guidelines advocate multifactorial fall prevention encompassing mobility assessment, sensory and cognitive evaluation, medication review, and modification of environmental hazards [2,26]. To ensure that older adults can reside safely in their own homes, robust fall reduction strategies are essential. Using an emergency call as an entry point to initiate on-site or follow-up fall prevention interventions and education represents a potential pathway. Repeated EMS use is common among older adults, supporting targeted prevention and follow-up after the initial encounter [27]. As falls are a major source of geriatric trauma and mortality, trauma centers can play an important role in prevention by coordinating collaborative efforts with emergency personnel and supporting continuity of care.

Among older adults, more than one in six EMS transports were followed by another transport of the same patient within 30 days [27]. Patients who are not transported to hospital after a fall constitute a particularly vulnerable group, exhibiting a high statistical risk of future falls and a substantial burden of chronic health conditions [28].

This patient group stands to benefit from integrated care pathways—including general practitioner consultations and community health resources—aimed at reducing the incidence of fall-related events. Expanding the development and implementation of such programs holds the potential to enhance patient well-being while simultaneously alleviating the strain on emergency departments and hospital infrastructure.

Fall-related emergencies constitute a significant burden on the emergency medical call system. Despite the clinical significance of the issue, large-scale research utilizing emergency response data to characterize geriatric falls and factors associated with hospital transfer in Türkiye is still lacking.

To the best of our knowledge, this is the first large-scale study in Türkiye to analyze emergency medical services data on falls among older adults. Several limitations should be noted. Cases were identified from the incident classification recorded by ambulance crews, so falls documented under another primary complaint would not have been captured, and the analysis may therefore underestimate the true number of EMS-attended falls. The health care facility location code combines falls occurring within a facility with records in which the individual had already reached a facility before EMS contact. Consequently, the recorded location may represent the point of EMS contact rather than the actual site of the fall. Because these situations cannot be distinguished within the available dataset, true falls occurring within health care facilities could not be isolated, and findings involving the non-residential category should therefore be interpreted with this limitation in mind. Because individual patient identifiers were unavailable, recurrent encounters by the same person could not be linked, and each EMS case record was treated as independent. The same individual may therefore have contributed more than one record; this potential within-person clustering could reduce statistical independence and affect the precision of the estimated confidence intervals. Because consciousness status was not included in the multivariable models, its independent association with either outcome could not be assessed after adjustment for the other covariates, and residual confounding related to neurological status cannot be excluded. The primary hospital-transfer outcome contrasted the specific EMS disposition code of hospital transfer with a heterogeneous group of other dispositions. Although exclusion of inter-hospital transfers in the sensitivity analysis did not alter the direction of the principal associations, the remaining reference categories still represent distinct operational and clinical circumstances and should not be considered equivalent. Important clinical characteristics, including frailty, functional status, comorbidity, and injury type, were not available in the dataset. Residual confounding by these and other unmeasured factors may therefore remain, and the adjusted estimates should be interpreted as associations rather than causal effects. The observation period ends in October 2025 rather than at the end of the calendar year, so annual counts are not directly comparable across the three years, and no inference about temporal trends in fall incidence should be drawn from them. Finally, the dataset captures only falls attended by EMS and therefore does not represent all falls occurring among older adults in the community.

## 5. Conclusions

In this nationwide analysis of 685,666 EMS-attended fall case records among adults aged ≥65 years, residential falls predominated, and their proportion increased with advancing age and was higher among women and during night and early-morning hours. In the adjusted fall-location model, higher odds of non-residential location were observed during daytime and evening hours and among men, whereas lower odds were observed with advancing age. Hospital transfer was most strongly associated with field triage category and fall location, and lower adjusted odds of hospital transfer were observed with advancing age, whereas higher odds were observed outside Istanbul. These findings describe variation in EMS-attended fall patterns and disposition across demographic, clinical, temporal, and broad geographic characteristics. Although preventive or referral interventions were not directly evaluated, the findings may inform future research on fall prevention, prehospital assessment, and referral pathways for older adults.

## Figures and Tables

**Table 1:** Characteristics of emergency medical services–attended fall case records among adults aged ≥65 years, Türkiye, January 2023–October 2025 (N = 685,666).

Variable	Group	f	%
Fall location	Residential	406,183	59.2
	Non-residential	279,483	40.8
Time of incident	00:00–07:59	81,730	11.9
	08:00–16:59	403,925	58.9
	17:00–23:59	200,011	29.2
Sex	Female	394,395	57.5
	Male	291,271	42.5
Age (years)	65–74	265,292	38.7
	75–84	263,131	38.4
	≥85	157,243	22.9
Consciousness	Normal	662,387	96.6
	Abnormal	23,279	3.4
Year	2023	239,617	34.9
	2024	253,634	37.0
	2025	192,415	28.1
City	Istanbul	101,566	14.8
	Non-Istanbul	584,100	85.2
Settlement type	Urban	523,272	76.3
	Rural	162,394	23.7
Triage category	Green	168,911	24.6
	Yellow	450,064	65.6
	Red	66,691	9.7

Abbreviation: f, frequency (number of case records). The unit of analysis is the emergency medical services (EMS) case record, not the unique patient. Consciousness status is an operational ambulance-crew assessment (normal = alert and oriented; abnormal = documented alteration in consciousness). Triage categories are operational field priority codes used by the 112 system and are not validated acuity scores. Records for 2025 cover January to October only; therefore, the lower record count for 2025 should not be interpreted as evidence of a decline in incidence.

**Table 2:** Case disposition by fall location.

Case Outcome	Residential, n	Residential, %	Non-Residential, n	Non-Residential, %	Total, n	Total, %
Death—transfer to morgue	40	0.01	84	0.03	124	0.02
Death—on scene	1289	0.3	605	0.2	1894	0.3
Transfer—other	2288	0.6	4933	1.8	7221	1.1
Transfer—home	2268	0.6	30,326	10.9	32,594	4.8
Transfer—inter-hospital	1623	0.4	81,451	29.1	83,074	12.1
Transfer—hospital	379,026	93.3	148,855	53.3	527,881	77.0
Transfer—refused	17,166	4.2	10,431	3.7	27,597	4.0
Transfer—medical examination	133	0.03	1890	0.7	2023	0.3
On-scene intervention	2238	0.6	815	0.3	3053	0.4
Other	112	0.03	93	0.03	205	0.03
Total	406,183	100.0	279,483	100.0	685,666	100.0

**Table 3:** Specific fall location within the residential and non-residential categories.

Residential Location	n	%	Non-Residential Location	n	%
Home	389,764	96.0	Health care facility	124,521	44.6
Nursing home	12,445	3.1	Street	114,456	41.0
Hotel	2925	0.7	Other	13,551	4.8
Prison	745	0.2	In a vehicle	7981	2.9
Dormitory	304	0.1	Highway	7509	2.7
			Field/terrain	7097	2.5
			Workplace	4368	1.6
Total	406,183	100.0	Total	279,483	100.0

The residential and non-residential categories are administrative location codes and do not correspond exactly to an indoor–outdoor distinction. The health care facility category includes both falls occurring within a health care facility and records in which the individual had already reached a facility before EMS contact; therefore, this category may reflect the point of EMS contact rather than the actual fall site.

**Table 4:** Fall location by patient, temporal, geographic, and triage characteristics.

Variable	Group	Residential, n (%)	Non-Residential, n (%)	*p*-Value	Cramér’s V
Time of incident	00:00–07:59	63,418 (77.6)	18,312 (22.4)	<0.001	0.142
	08:00–16:59	224,219 (55.5)	179,706 (44.5)		
	17:00–23:59	118,546 (59.3)	81,465 (40.7)		
Age (years)	65–74	131,505 (49.6)	133,787 (50.4)	<0.001	0.171
	75–84	162,933 (61.9)	100,198 (38.1)		
	≥85	111,745 (71.1)	45,498 (28.9)		
Sex	Female	258,109 (65.4)	136,286 (34.6)	<0.001	0.147
	Male	148,074 (50.8)	143,197 (49.2)		
City	Istanbul	62,965 (62.0)	38,601 (38.0)	<0.001	0.023
	Non-Istanbul	343,218 (58.8)	240,882 (41.2)		
Triage category	Green	96,049 (56.9)	72,862 (43.1)	<0.001	0.031
	Yellow	271,423 (60.3)	178,641 (39.7)		
	Red	38,711 (58.0)	27,980 (42.0)		
Settlement type	Urban	305,325 (58.3)	217,947 (41.7)	<0.001	0.033
	Rural	100,858 (62.1)	61,536 (37.9)		
Consciousness	Normal	392,998 (59.3)	269,389 (40.7)	<0.001	0.010
	Abnormal	13,185 (56.6)	10,094 (43.4)		

Percentages are row percentages. *p*-values are from Pearson chi-square tests. Cramér’s V is reported as a measure of association strength. Cohen-derived benchmarks were applied according to degrees of freedom (df): 0.10/0.30/0.50 for df = 1 and 0.07/0.21/0.35 for df = 2, corresponding to small/medium/large associations [17].

**Table 5:** Multivariable logistic regression model for non-residential fall location.

Variable	Group	OR	95% CI, Lower	95% CI, Upper	*p*-Value
Time of incident	00:00–07:59	Reference			
	08:00–16:59	2.871	2.820	2.923	<0.001
	17:00–23:59	2.390	2.345	2.437	<0.001
Triage category	Green	Reference			
	Yellow	0.897	0.886	0.907	<0.001
	Red	0.939	0.921	0.957	<0.001
Age (years)	65–74	Reference			
	75–84	0.624	0.617	0.631	<0.001
	≥85	0.424	0.418	0.430	<0.001
Sex	Female	Reference			
	Male	1.761	1.743	1.778	<0.001
City	Istanbul	Reference			
	Non-Istanbul	1.140	1.124	1.157	<0.001
Settlement type	Urban	Reference			
	Rural	0.793	0.784	0.803	<0.001

Abbreviations: CI, confidence interval; OR, odds ratio. Outcome: non-residential versus residential fall location. An OR greater than 1 indicates higher odds of a non-residential location relative to the reference category.

**Table 6:** Multivariable logistic regression model for hospital transfer.

Variable	Group	OR	95% CI, Lower	95% CI, Upper	*p*-Value
Time of incident	00:00–07:59	Reference			
	08:00–16:59	0.959	0.931	0.988	0.006
	17:00–23:59	1.124	1.088	1.161	<0.001
Fall location	Residential	Reference			
	Non-residential	0.268	0.264	0.273	<0.001
Triage category	Green	Reference			
	Yellow	11.063	10.858	11.271	<0.001
	Red	8.053	7.780	8.337	<0.001
Age (years)	65–74	Reference			
	75–84	0.863	0.846	0.880	<0.001
	≥85	0.739	0.722	0.756	<0.001
Sex	Female	Reference			
	Male	1.158	1.138	1.179	<0.001
City	Istanbul	Reference			
	Non-Istanbul	1.999	1.953	2.045	<0.001
Settlement type	Urban	Reference			
	Rural	1.165	1.140	1.191	<0.001

Abbreviations: CI, confidence interval; OR, odds ratio. Outcome: hospital transfer versus all other case dispositions. An OR greater than 1 indicates higher odds of hospital transfer relative to the reference category. Because hospital transfer occurred in 77.0% of case records, ORs should not be interpreted as risk ratios or as equivalent relative changes in absolute probability. The outcome denotes the specific administrative EMS disposition category “hospital transfer” as operationally defined in Section 2.5.

## Data Availability

Restrictions apply to the availability of these data. The data were obtained from the Emergency Health Automation System of the Republic of Türkiye Ministry of Health and are not publicly available. Requests to access the datasets should be directed to the General Directorate of Emergency Health Services of the Ministry of Health and are subject to authorization.

## Supplementary Materials

The following supporting information can be downloaded at https://www.mdpi.com/article/10.3390/jcm15187239/s1, Supplementary File S1: STROBE checklist for cross-sectional studies.
