# Supplementary material for: Falls Among Older Adults in Türkiye: A Nationwide Analysis of Emergency Medical Service Data
Source: J Clin Med. 2026 Sep 17;15(18):7239. doi: 10.3390/jcm15187239 (PMC13607967; doi:10.3390/jcm15187239)
Supplement: Supplementary file 1 [file jcm-15-07239-s001.zip › jcm-4541315-supplementary.pdf]

Supplementary File S1

Completed STROBE Checklist for Cross-Sectional Studies

Manuscript: Falls Among Older Adults in Türkiye: A Nationwide Analysis of Emergency Medical Services Data

Note. Checklist structure adapted from the STROBE reporting recommendations for cross-sectional studies. Page references correspond to the clean revised manuscript generated from the current revision; section, table, and figure identifiers are also provided to preserve traceability if pagination changes during production. “Not applicable” is used where the corresponding analysis was not performed or the item does not apply to this administrative-record study.

Section	Item	STROBE recommendation (adapted summary)	Location in revised manuscript / reporting note
**Title and abstract**	1(a)	Identify the observational study design using a standard design term in the title or abstract.	p. 1 (Abstract: retrospective analysis); p. 2 (Section 2.1: retrospective, observational, national case-level analysis).
Title and abstract	1(b)	Provide an informative, balanced abstract summarizing what was done and the principal findings.	p. 1 (structured Abstract: Background/Objectives, Methods, Results, Conclusions).
**Introduction**	2	Explain the scientific background and rationale for the investigation.	pp. 1–2 (Introduction).
Introduction	3	State the specific study objectives and any prespecified hypotheses, if applicable.	p. 2 (final paragraph of Introduction; no prespecified hypothesis stated).
**Methods**	4	Present the key elements of the study design early in the manuscript.	p. 2 (Section 2.1, Study Design and Setting).
Methods	5	Describe the setting, locations, and relevant dates/period of data collection.	p. 2 (Section 2.1: nationwide 112 EMS, all 81 provinces, January 2023–October 2025; 34 months).
Methods	6(a)	State eligibility criteria and explain the source and method used to select records/participants.	pp. 2–3 (Sections 2.2–2.3; adults aged ≥65 years with EMS records coded as falls).
Methods	7	Define outcomes, exposures/predictors, potential confounders, effect modifiers, and diagnostic criteria where relevant.	pp. 3–4 (Section 2.4, Variables and Definitions; Section 2.5, Outcome Definition).
Methods	8*	For each variable, describe the data source and method of assessment/measurement; address comparability across groups where relevant.	pp. 2–4 (Section 2.2 data source; Section 2.4 operational EMS variable definitions and coding).
Methods	9	Describe efforts used to address potential sources of bias.	pp. 3–5 (complete-data inclusion, operational definitions, sensitivity analysis excluding inter-hospital transfers); pp. 10–11 (limitations addressing misclassification, repeated encounters, residual confounding, and generalizability).
Methods	10	Explain how the study size was determined.	p. 3 (Section 2.3 and Figure 1: 1,514,780 fall-related EMS records across all ages; 829,114 aged <65 excluded; final n = 685,666).
Methods	11	Explain how quantitative variables were handled, including any categorization and the rationale for chosen groups.	pp. 3–4 (Section 2.4: time categories and age groups 65–74, 75–84, ≥85 years, with rationale).
Methods	12(a)	Describe all statistical methods, including approaches used to control for confounding.	pp. 4–5 (Section 2.6: descriptive analyses, chi-square tests, Cramér’s V, and two multivariable logistic regression models).
Methods	12(b)	Describe methods used for subgroup or interaction analyses.	Not applicable. No subgroup or interaction analyses were performed; the Discussion explicitly notes that no sex-by-location interaction or location-stratified model was fitted (p. 10).
Methods	12(c)	Explain how missing data were handled.	p. 3 (Section 2.3: no missing values in study variables among age-eligible records; no imputation required); p. 5 (no observations dropped from either multivariable model).

Section	Item	STROBE recommendation (adapted summary)	Location in revised manuscript / reporting note
Methods	12(d)	If relevant, describe analytical methods accounting for the sampling strategy.	Not applicable. The analysis used national administrative EMS case records meeting the inclusion criteria; no sampling weights or complex survey design methods were used.
Methods	12(e)	Describe any sensitivity analyses.	pp. 4–5 (Sections 2.5–2.6: inter-hospital transfers excluded; hospital-transfer model refitted in n = 602,592).
**Results**	13(a)	Report the number of records/participants at each stage of study selection and analysis.	p. 3 (Section 2.3 and Figure 1: 1,514,780 identified; 829,114 aged <65 excluded; 685,666 eligible/analyzed; missing-data exclusions = 0).
Results	13(b)	Give reasons for non-participation/exclusion at each stage.	p. 3 (age <65 years was the prespecified exclusion; no exclusions for missing study variables).
Results	13(c)	Consider presenting a flow diagram.	p. 3 (Figure 1, study flow).
Results	14(a)	Report participant/record characteristics and information on relevant exposures/predictors/confounders.	pp. 5–7 (Table 1; Tables 3–4).
Results	14(b)	Report the amount of missing data for each variable of interest.	p. 3 (Section 2.3: no missing values for age, sex, fall location, triage category, transfer status, consciousness status, settlement type, or city); p. 5 (Table 1; n = 685,666).
Results	15*	Report outcome-event numbers or summary measures.	pp. 5–6 (residential/non-residential fall counts; Table 2 disposition counts, including hospital transfer 527,881 [77.0%]).
Results	16(a)	Report unadjusted estimates and, where applicable, adjusted estimates with measures of precision; state the variables included in adjustment.	pp. 6–8 (Table 4: unadjusted distributions and Cramér's V; Tables 5–6: adjusted ORs with 95% CIs). Covariates and reference categories are specified in Section 2.6 (pp. 4–5).
Results	16(b)	Report category boundaries when continuous variables are categorized.	pp. 3–4 (Section 2.4) and pp. 5–8 (Tables 1, 4–6: age 65–74, 75–84, ≥85 years; time 00:00–07:59, 08:00–16:59, 17:00–23:59).
Results	16(c)	If relevant, consider translating relative-risk estimates into absolute risk over a meaningful period.	Not applicable. The multivariable models report odds ratios, not risk ratios; because hospital transfer was common, the manuscript explicitly avoids interpreting ORs as risk ratios or equivalent changes in absolute probability (pp. 4–5; Table 6, p. 8).
Results	17	Report other analyses, such as subgroup, interaction, or sensitivity analyses.	p. 8 (sensitivity analysis excluding 83,074 inter-hospital transfer records; n = 602,592; principal estimates reported). No subgroup or interaction analyses were performed.
**Discussion**	18	Summarize the key results in relation to the study objectives.	p. 8 (opening paragraph of Discussion) and p. 11 (Conclusions).
Discussion	19	Discuss study limitations, including potential sources of bias or imprecision and their likely implications.	pp. 10–11 (limitations: incident classification, health-care-facility location misclassification, repeated encounters, consciousness exclusion, heterogeneous disposition reference group, residual confounding, incomplete 2025 calendar year, EMS-only capture).
Discussion	20	Provide a cautious overall interpretation considering objectives, limitations, multiple analyses, related studies, and other relevant evidence.	pp. 8–11 (Discussion; findings compared with prior literature and interpreted as associations rather than causal effects).
Discussion	21	Discuss generalizability/external validity.	pp. 2–4 (national EMS setting and broad geographic classification) and pp. 10–11 (limitation that EMS-attended falls do not represent all community falls).
**Other information**	22	Report the funding source and, when applicable, the role of the funder.	p. 11 (Funding: "This research received no external funding." No funder role applicable).

* Where the STROBE item requests information separately by comparison group, the manuscript reports residential and non-residential groups separately in the relevant tables.

Reference: von Elm E, Altman DG, Egger M, et al. The Strengthening the Reporting of Observational Studies in Epidemiology (STROBE) statement: guidelines for reporting observational studies. J Clin Epidemiol. 2008;61(4):344–349. doi:10.1016/j.jclinepi.2007.11.008.
